# Characterization of a novel pyruvate kinase from *Trichinella spiralis* and its participation in sugar metabolism, larval molting and development

**DOI:** 10.1371/journal.pntd.0010881

**Published:** 2022-10-31

**Authors:** Wen Wen Yue, Shu Wei Yan, Ru Zhang, Yong Kang Cheng, Ruo Dan Liu, Shao Rong Long, Xi Zhang, Zhong Quan Wang, Jing Cui

**Affiliations:** Department of Parasitology, Medical College, Zhengzhou University, Zhengzhou, People’s Republic of China; University of Liverpool, UNITED KINGDOM

## Abstract

**Background:**

Pyruvate kinase widely exists in many parasites and plays an important role in the energy production for the parasites. Pyruvate kinase might be a potential drug target for killing the parasites. The aim of the present study was to evaluate the biological characteristics and roles of *T*. *spiralis* pyruvate kinase M (TsPKM) in sugar metabolism, larval molting and development of *T*. *spiralis*.

**Methodology/Principal findings:**

TsPKM has two functional domains of pyruvate kinase and the tertiary structure of TsPKM is tetramer which has the enzyme active site constituted by 8 amino-acid residues (Arg71, Asn73, Asp110, Phe241, Lys267, Glu269, Asp293 and Thr325). Recombinant TsPKM (rTsPKM) was expressed and purified. The rTsPKM had good immunogenicity. RT-PCR and Western blot showed that TsPKM was transcribed and expressed at various developmental stages in *T*. *spiralis* lifecycle. Immunofluorescence test showed that TsPKM was principally located in the cuticle, muscle, stichosome, intestine and the intrauterine embryos of female adults. rTsPKM catalyzed the reaction of phosphoenolpyruvate (PEP) and adenosine diphosphate (ADP) to produce pyruvic acid and adenosine triphosphate (ATP). TsPKM played an important role in the metabolism and energy production of *T*. *spiralis*. After silencing of TsPKM gene by specific dsRNA-TsPKM2, protein expression and enzyme activity of TsPKM decreased by 50.91 and 26.06%, respectively. After treatment with RNAi, natural TsPKM enzyme activity, larval molting, sugar metabolism, growth and development of *T*. *spiralis* were significantly reduced.

**Conclusions:**

TsPKM participates in the larval molting, sugar metabolism, growth and development of *T*. *spiralis* and it might be a candidate target of therapeutic drug of trichinellosis.

## Author summary

Pyruvate kinases belong to transferases and can transfer the high-energy phosphate bond of phosphoenolpyruvate (PEP) to adenosine diphosphate (ADP) to produce pyruvic acid and adenosine triphosphate (ATP). Pyruvate kinases play a significant biological role in the parasite survival in hosts. Our results revealed that TsPKM was expressed at various *T*. *spiralis* developmental stages, and principally located in the cuticle, stichosome, intestine and the intrauterine embryos of female adults. rTsPKM catalyzed the reaction of phosphoenolpyruvate (PEP) and adenosine diphosphate (ADP) to produce pyruvic acid and adenosine triphosphate (ATP). TsPKM played an important role in the metabolism and energy production of *T*. *spiralis*. Protein expression and enzyme activity of TsPKM were decreased by 50.91 and 26.06% respectively through silencing of TsPKM gene using specific dsRNA-TsPKM2. After treatment with RNAi and inhibitor tannin, natural TsPKM activity, larval molting, sugar metabolism, growth and development of *T*. *spiralis* were obviously inhibited. Our results showed that TsPKM participates in *T*. *spiralis* molting, sugar metabolism and development, and it might be a candidate target for anti-*Trichinella* drugs.

## Introduction

*Trichinella spiralis* is a tissue-parasiting nematode of the genus *Trichinella* that causes trichinellosis. Trichinellosis is a zoonotic parasitic disease caused by eating raw or semi-raw meat containing the encapsulated infective muscle larvae (ML), which is widely prevalent all over the world [1]. From 2009 to 2020, there were 8 outbreaks of human trichinellosis in China, which consisted of 479 cases and 2 deaths [2]. Pork is the predominant source of *T*. *spiralis* infection in humans. *Trichinella* infection is not only an important public health problem but also a tremendous threat to meat food safety [3–5].

After being ingested, the encapsulated ML in infected meat are released from their capsules under the help of gastric digestive enzymes and activated into intestinal infectious larvae (IIL) by bile, the IIL invade into enteral epithelium and undergo molting four times to develop into adult worms (AW) [6,7]. The female AW produce newborn larvae (NBL) that circulates via the lymph to bloodstream and invade the skeletal muscles to develop into the encapsulated ML for completing their lifecycle [8]. *T*. *spiralis* mainly obtains various energy required for its survival through glucose metabolism, and generally has a complete citric acid cycle [9]. Hexokinase, phosphofructokinase-1 and pyruvate kinase are the three key enzymes in glycolysis pathway. Pyruvate kinase belongs to transferases, and it can catalyze the reaction of phosphoenolpyruvate (PEP) and adenosine diphosphate (ADP) to produce pyruvic acid and adenosine triphosphate (ATP). ATP produced by catalysis provides energy for the organisms. Pyruvic acid participates in the subsequent aerobic and anaerobic oxidation to provide energy for the life activities of the organisms. At the same time, it completes the conversion of sugar, fat and amino acids under the action of acetyl coenzyme A and citric acid cycle [10]. Glycolysis is a common pathway of anaerobic and aerobic oxidation of sugar, which is from the phosphorylation of glucose to the final production of Pyruvic acid. All factors that affect glycolysis also affect the anaerobic and aerobic oxidation of sugar, thus affecting the utilization of glucose, the synthesis of ATP and the conversion of three nutrients. Therefore, pyruvate kinase plays an important role in cell metabolism.

Pyruvate kinase widely exists in some free-living nematode and parasites, such as *Caenorhabditis elegans* [11], *Schistosoma japonicum* [12], cestode [13], *Giardia lamblia* [14], *Plasmodium* [15], *Toxoplasma gondii* [16] and *Cryptosporidium* [17]. It plays an important role in the energy production for the parasites and the nutrient conversion, and then affects the growth, development and reproduction of parasites. Due to the important role of pyruvate kinase in cell metabolism, the energy of parasites can be exhausted by inhibiting or knocking out the expression of pyruvate kinase gene, which can lead to parasite death. Therefore, pyruvate kinase is a potential drug target for killing the parasites.

In this study, a novel *T*. *spiralis* pyruvate kinase M (TsPKM, GenBank: KRY30732.1) was acquired from *T*. *spiralis* draft genome [18]. In mammals, pyruvate kinase was divided into three different subtypes, namely PKL, PKR and PKM (PKM1 and PKM2). PKL is mainly expressed in liver, kidney and intestine; PKR is expressed in red blood cells, and PKM is principally expressed in tissues with high energy metabolism and rapidly proliferating cells [19]. The pyruvate kinase from *T*. *spiralis* is assigned to the PKM subtype. The aim of the present study was to evaluate the biological characteristics and roles of TsPKM in sugar metabolism, larval molting and development of *T*. *spiralis*.

## Materials and methods

### Ethics statement

This study was performed in the light of National Guidelines for Experimental Animal Welfare (Minister of Science and Technology, People’s Republic of China, 2006). All animal experiments in the current research were approved by the Life Science Ethics Committee of Zhengzhou University (No. SCXK 2020–0004).

### Parasites, cells and experimental animals

The species of *Trichinella spiralis* (ISS534) was collected from a naturally infected domestic pig in central China [20], and passaged in BALB/c mice in our laboratory. Intestinal epithelium cells (IECs) were isolated from small intestine of normal fetal mice [21,22]. Female BALB/c mice aged 4–6 weeks were purchased from Henan Provincial Experimental Animal Center.

### Worm collection and protein preparation

The ML were collected by artificially digestion of *T*. *spiralis-*infected mouse muscles at 42 days post infection (dpi) [23]. The IIL were recovered from the intestine of infected mice at 6 hours post infection (hpi) [24,25]. The AW at 3 and 6 dpi were also collected from infected murine small intestine [26]. The adult females at 6 dpi were cultured in RPMI-1640 with 10% fetal bovine serum (FBS; Gibco) at 37°C in 5% CO_2_ for 24 h, and the NBL were harvested as previously described [27]. Worm soluble proteins of various stage worms (ML, IIL, AW and NBL), excretion and secretion (ES) proteins from ML, IIL and AW were prepared as described previously [28,29]. Briefly, various *T*. *spiralis* stages (ML, IIL and AW) were washed using sterile saline, cultured in RPMI-1640 medium (5000 worms/ml) at 37°C, 5% CO_2_ for 18 h. The culture medium containing ES proteins was filtered by a 0.22 μm membrane, and concentrated using an ultrafiltration tube. The concentration of ES proteins was assayed by a Coomassie brilliant blue G-250 method [26].

### Bioinformatics analysis of TsPKM

The full-length cDNA sequence of TsPKM gene was retrieved from GenBank (GenBank: KRY30732.1) [30]. The physicochemical properties and biological characteristics were analyzed through the online bioinformatics website. Multiple sequence alignment of the amino acid sequence of pyruvate kinase among different species of the genus *Trichinella* was compared by Cluster Omega [31,32]. The GenBank accession numbers of pyruvate kinase from other *Trichinella* species and organisms were as follows: *Trichinella* T8 (KRZ85571.1), *Trichinella* T6 (KRX84774.1), *T*. *murrelli* (KRX42370.1), *T*. *nativa* (OUC39704.1), *T*. *britovi* (KRY55915.1), *T*. *patagoniensis* (KRY12085.1), *T*. *nelsoni* (KRX15479.1), *T*. *pseudospiralis* (KRX90766.1), *T*. *papuae* (KRZ80248.1), *T*. *zimbabwensis* (KRZ04871.1), *Homo sapiens* (AAA60104.1) and *Mus musculus* (BAA23642.1). The phylogenetic tree was constructed using MEGA 7.0 on the basis of the Neighbor-joining (NJ) method as described before [33,34].

### Cloning and expression of rTsPKM and preparation of anti-rTsPKM serum

Total RNA was extracted from the ML using Trizol (Invitrogen, USA), reversely transcribed into the cDNA and used as a template to amplify the TsPKM gene. *BamH* I and *Pst* I (**Bold**) were selected as restriction sites to design TsPKM-specific primers (5’-CGC**GGATCC**ATGTCCGAAAAGCAAAGTCAGAAGA-3’, 5’-AA**CTGCAG**TCATGGTCTTGGAATTAGAGGTTCG-3’) [35]. The full length TsPKM cDNA sequence was amplified by PCR. The PCR products were cloned into the expression vector pQE-80L with a His-tag at N-terminus (Novagen, USA), and recombinant pQE-80L/TsPKM was introduced into *E*. *coli* BL21 (Novagen). The expression of rTsPKM was induced at 25°C for 23 h using 0.2 mM IPTG [36], rTsPKM was purified by nickel column affinity chromatography (Sangon Biotech, Shanghai, China) [37]. Expression of rTsPKM protein was analyzed by SDS-PAGE and Western blotting as described before [38].

Ten mice were immunized subcutaneously with 20 μg rTsPKM mixed with complete Freund’s adjuvant. Boost immunization was administered three times with 20 μg rTsPKM mixed with incomplete Freund’s adjuvant at a 2-week interval [39]. Two weeks after the last immunization, tail blood of immunized mice was collected to isolate anti- rTsPKM immune sera, the IgG antibody titer of anti-rTsPKM serum was measured by ELISA [40,41].

### SDS-PAGE and Western blotting analysis

Soluble worm somatic crude and ES proteins of diverse *T*. *spiralis* phases, and purified rTsPKM were separated on 10% SDS-PAGE [24,42]. The proteins were transferred onto nitrocellulose (NC) membrane (Millipore, USA) in the semi-dry transfer cell (Bio-Rad, USA) [43]. The membrane was blocked with 5% skimmed milk in Tris-buffered saline containing 0.05% Tween (TBST) at 37°C for 2 h, and cut into strips. The strips were probed by various sera (1:100; anti-rTsPKM serum, infection serum and pre-immune serum) at 37°C for 2 h. After washes with TBST, the strips were incubated at 37°C for 1 h with HRP-anti-mouse IgG conjugate (1:10000; Southern Biotech, USA). After being washed again, the strips were colored using 3, 3’-diaminobenzidine tetrahydrochloride (DAB; Sigma-Aldrich) or an enhanced chemiluminescent kit (ECL, Solarbio, China) [44,45].

### RT-PCR analysis of TsPKM transcription in different *T*. *spiralis* phases

Total RNAs from diverse *T*. *spiralis* stages (ML, IIL, 3 d AW and NBL) were isolated using Trizol (Invitrogen, USA) [46,47]. RT-PCR was performed to evaluate the TsPKM mRNA expression in diverse *T*. *spiralis* stages as previously described [48]. A *T*. *spiralis* housekeeping gene GAPDH (GenBank: AF452239) was also amplified and used as an internal control [49,50]. Each experiment had three replicates.

### Immunofluorescence test (IFT)

The fresh whole worms of different *Trichinella spiralis* stages (ML, IIL, 3 d AW and NBL) were fixed with 4% paraformaldehyde for 30 min and embedded in paraffin and cut into a 2-μm thick cross-section with a microtome. Expression and worm tissue localization of natural TsPKM in diverse worm stages were investigated using the IFT technique as reported before [43,51]. Briefly, the whole worms and cross-sections were first blocked with 5% goat serum at 37°C for 2 h, and then incubated at 37°C for 2 h with various sera (1: 10 dilutions of anti-rTsPKM serum, infection serum and normal serum). After washes with PBS, the worms and cross-sections were incubated with FITC-conjugated anti-mouse IgG (1:100; Abways, Shanghai, China). After washes again, they were observed under fluorescent microscopy (Olympus, Tokyo, Japan) [52,53].

### Enzyme activity analysis of rTsPKM

The enzyme activity of rTsPKM was assayed by using 2,4-dinitrophenylhydrazine method. The reaction mixture composition and reaction conditions are as follows. rTsPKM (8 μl) was first added into matrix solution (32 μl; 2.5 mM PEP, 1.25 mM ADP, pH 8.0 Tris-HCl), and incubated at 37°C for 10 min. The reaction was terminated with incubation of 80 μl of reaction stop solution (0.0625 g 2,4-dinitrophenylhydrazine, 25 ml of 10 mol/L HCl, 250 ml distilled water) at 37°C for 10 min. Coloration was developed with 8 μl of coloration solution (730.6 mg EDTA, 100 ml distilled water, 2.5 mol/L NaOH 400 ml). Finally, a microplate reader was used to measure the absorbance at 510 nm. The *in vitro* optimum catalytic conditions of rTsPKM were determined by changing the concentration of rTsPKM, reaction temperature and pH of buffer solution [54]. In order to verify whether rTsPKM enzymatic activity is metal ion-dependent, seven common auxiliary metal ions (Fe^2+^, Mn^2+^, Ca^2+^, Co^2+^, Ni^2+^, Cu^2+^ and Zn^2+^) were added into the reaction system at the same concentration (0.3 mM) to analyze their effect on rTsPKM activity [49,55]. Previous studies showed that ethyl pyruvate had little side effects on humans and was easy to penetrate the blood brain barrier [56]. Therefore, ethyl pyruvate was also used as the enzyme inhibitor of TsPKM in the current study. Different concentrations of inhibitors (tannin, ethyl pyruvate) and common protease inhibitors 1,10-phenanthroline (1 mM), E64 (5 μM), EDTA (10 mM) and PMSF (1 mM) were added to the reaction system to determine the effects of different inhibitors on rTsPKM enzyme activity [57]. Under the optimum catalytic conditions of rTsPKM enzyme activity, the corresponding enzyme kinetic parameters were determined by detecting the reaction of ADP with various concentrations of PEP, and the reaction of PEP with different concentrations of ADP [28].

### Suppression of inhibitors on native TsPKM activity in worm somatic proteins

Total of 2000 ML were first incubated with at 37°C for 2 hours with various doses of tannin (25, 50, 75 and 100 μM) or ethyl pyruvate (10, 20, 30, 40 mM) [56,58]. After being washed with PBS for 3 times, the larval soluble proteins were prepared [59]. The enzyme activity of native TsPKM in ML somatic proteins was measured by 2,4-dinitrophenylhydrazine method.

### RNA interference (RNAi)

According to the cDNA sequence of TsPKM, three pairs of TsPKM-specific primers containing T7 promoter and enhancer were designed (5’-GATCAC**TAATACGACTCACT ATAGGG**ATGTCCGAAAAGCAAAGT-3’, 5’-GATCAC**TAATACGACTCACTATAGGG** CGTCACCCTCACAGCGCTGTT-3’. 5’-GATCAC**TAATACGACTCACTATAGGG**GATA CCAAAGGGCCCGAAAT-3’, 5’-GATCAC**TAATACGACTCACTATAGGG**TTCGATTTT GGCCACA-3’. 5’-GATCAC**TAATACGACTCACTATAGGG**GTCAATTTGCCAGACT GT-3’, 5’-GATCAC**TAATACGACTCACTATAGGG**GTCTGCACCGTCCAGTAC-3’) [27,60]. Green fluorescent protein (GFP) was selected as the control, and its primers were as follows: 5’-GATCAC**TAATACGACTCACTATAGGG**TCCTGGTCGAGCTGGACGG-3’, 5’-GATCAC**TAATACGACTCACTATAGGG**CGCTTCTCGTTGGGGTCTTTG-3’. The dsRNA-TsPKM and dsRNA-GFP were transfected into the ML by electroporation (200 V 25 μF 200 Ω) and cultured in RPMI-1640 for 3 days [61]. Transcription and expression levels of TsPKM gene in the ML were assessed by qPCR and Western blotting as described previously [62,63].

### Effects of RNAi on the *in vitro* larval glycometabolism

Total 2000 ML were transfected with 60 ng/μl dsRNA-TsPKM2, dsRNA-GFP and PBS, respectively, and cultured for 3 days. Besides, 2000 ML were treated with 100 μM tannin for 2 hours. Larval ATP contents from different groups were measured by ATP assay kit (Sangon Biotech, Shanghai, China). Moreover, the glycogen distribution in worm tissues was observed on larval sections using periodic acid-schiff stain (PAS; Baso, Zhuhai, China) [33,64]. The distribution of lipid droplets in whole intact ML was examined using oil red O staining [65]. Furthermore, the larval soluble somatic proteins were prepared, and total sugar and lipid content in larval soluble proteins was measured by anthrone-sulfuric method and acetylacetone method, respectively [66,67].

### The *in vitro* larval molting test

The IIL are divided into four stages according to the molting time; include intestinal infective L_1_ larvae (IIL1, 0.9 h after infection), L_2_ larvae (10–14 h), L_3_ larvae (15–22 h) and L_4_ larvae (23–30 h) [62]. To assess the suppressive role of dsRNA-TsPKM and tannin on the larval molting, the *in vitro* invasion of mouse intestinal epithelial cells (IEC) was performed as previously described [42,68]. Briefly, ML was first activated into IIL1 using 5% swine bile at 37°C for 2 h. After washes with saline solution, the IIL larvae were treated by dsRNA-TsPKM or tannin, respectively. After treatment, fifty IIL were added to the semi-solid medium (DMEM+1.75% agarose) on an IEC monolayer and cultured in 37°C 5% CO_2_ for 3 days [69]. Each group had triplicates. The larval molting was observed and counted under a light microscope [45].

### Effects of RNAi on the *in vivo* larval development and glycometabolism

In order to further verify the TsPKM role in glycometabolism and development in *T*. *spiralis* life cycle, 100 mice were randomly divided into 5 groups (20 animals per group). Each mouse was orally with 500 ML treated with 60 ng/μl dsRNA-TsPKM2, dsRNA-GFP, PBS, tannin, or saline. All infected mice were euthanized at 24 h and 3 dpi, 24 h IIL (L4 larvae) and 3 d AW were recovered and numbered from small intestine of infected mice as reported before [70,71]. The morphology, worm length from various groups of infected mice were observed and measured under microscopy [72]. Native TsPKM enzyme activity, sugar and lipid content of various stage worms from infected mice were also ascertained as before.

### Statistical analysis

The data of this study were analyzed by SPSS 21.0 software and shown as arithmetic mean ± SD (standard deviation). One-way ANOVA and Student’s t test were used to analyze the difference in relative TsPKM mRNA transcription, protein expression, enzyme activity, larval glycolipid content, worm burdens and length. The differences of larval molting rate among various groups were analyzed using Chi-square test. Statistical difference level was *P* < 0.05.

## Results

### Bioinformatics analysis of TsPKM

**Fig 1:**
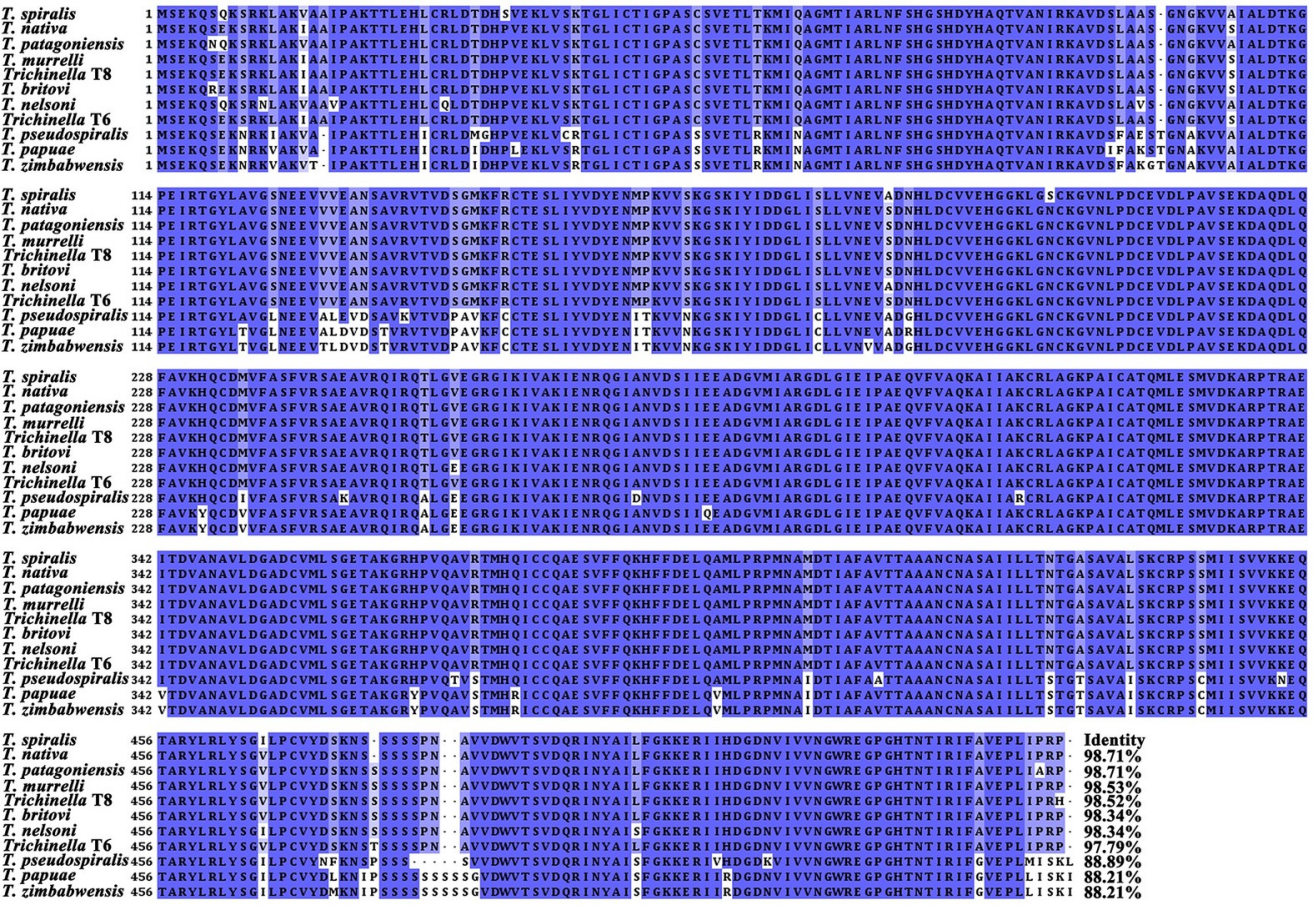
Multi sequence alignment of pyruvate kinase of different species/genes of *Trichinella*. According to the analysis of Cluster Omega, the same amino acids are marked in blue and conservative substitution of amino acid residues are marked in light blue. The pyruvate kinase genes of different species/genotypes of *Trichinella* have a high homology. The number at the end of each sequence represents the percentage of identity with TsPKM.

**Fig 2:**
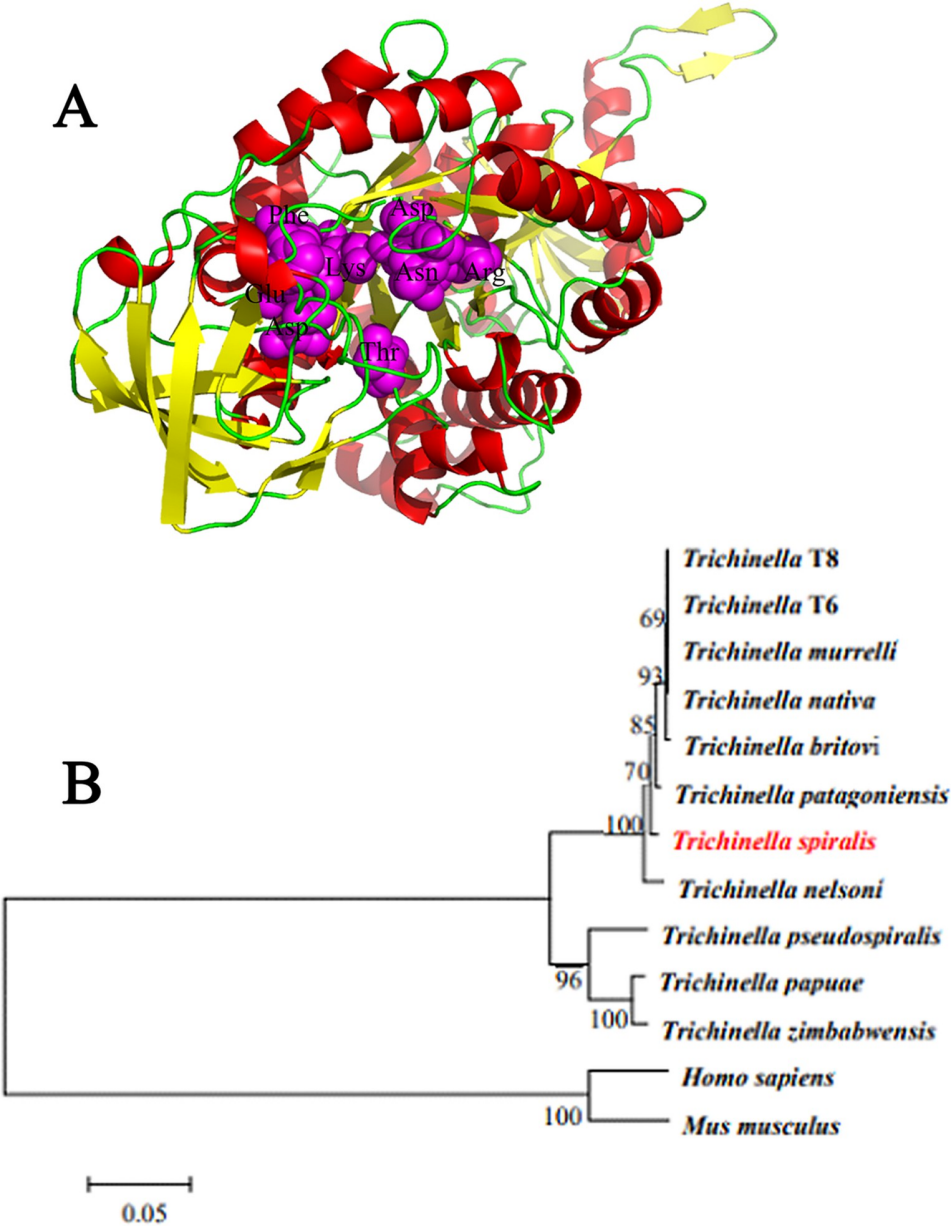
Tertiary structure prediction of TsPKM and evolutionary tree construction of pyruvate kinase of 13 organisms. **A:** Tertiary structure prediction of TsPKM. Eight amino-acid residues (Arg, Asn, Asp, Phe, Lys, Glu, Asp and Thr) constitute the enzyme active site signed as purple. **B:** TsPKM in the evolutionary tree of *Trichinella*, human and mouse. The evolutionary tree of pyruvate kinase of 11 different species/genotypes of the genus *Trichinella* was constructed by neighbor-joining (NJ) method. The encapsulated and non-encapsulated *Trichinella* was localized in two different evolutionary clades of *Trichinella*.

The complete cDNA sequence of TsPKM has a full length of 1629 bp and encodes 542 amino acids. The predicted molecular weight is 58.48 kDa and pI is 7.16. The TsPKM has obvious hydrophilicity at the N-terminus, and doesn’t have transmembrane region and signal peptide. The amino acid sequences of the TsPKM had an identity of 98.71, 98.71, 98.53, 98.52, 98.34, 98.34 and 97.79% with pyruvate kinase of the 7 encapsulated *Trichinella* species/genotypes (*T*. *nativa*, *T*. *patagoniensis*, *T*. *murrelli*, *Trichinella* T8, *T*. *nelsoni*, *T*. *britovi*, and *Trichinella* T6), and it had an identity of 88.89, 88.21 and 88.21% with pyruvate kinase from 3 non-encapsulated *Trichinella* species (*T*. *pseudospiralis T*. *papuae* and *T*. *zimbabwensis*,) (Fig 1). TsPKM belonged to transferases. It had two functional domains of pyruvate kinase, and secondary structure had 25 α-helixes, 32 β-strand, 18 β-turns, and 15 irregular coils. The tertiary structure analysis of TsPKM showed that the enzyme active site of TsPKM was constituted by 8 amino-acid residues (Arg71, Asn73, Asp110, Phe241, Lys267, Glu269, Asp293 and Thr325) (Fig 2A). The enzyme active site of TsPKM was highly conserved in different species of the genus *Trichinella* and also is the binding site of the metal ions (K^+^ and Mg^2+^) and substrate. The phylogenetic tree revealed that a monophyletic group of the genus *Trichinella* was well supported. Within the genus *Trichinella*, two clear clades were shown: one was the clade of 8 encapsulated species/genotypes (*T*. *spiralis*, *T*. *nativa*, *T*. *patagoniensis*, *T*. *murrelli*, *Trichinella* T8, *T*. *nelsoni*, *T*. *britovi*, and *Trichinella* T6), and the other was the clade of three non-encapsulated species (*T*. *pseudospiralis*, *T*. *papuae* and *T*. *zimbabwensis*) (Fig 2B).

### Expression and antigenic identification of rTsPKM

**Fig 3:**
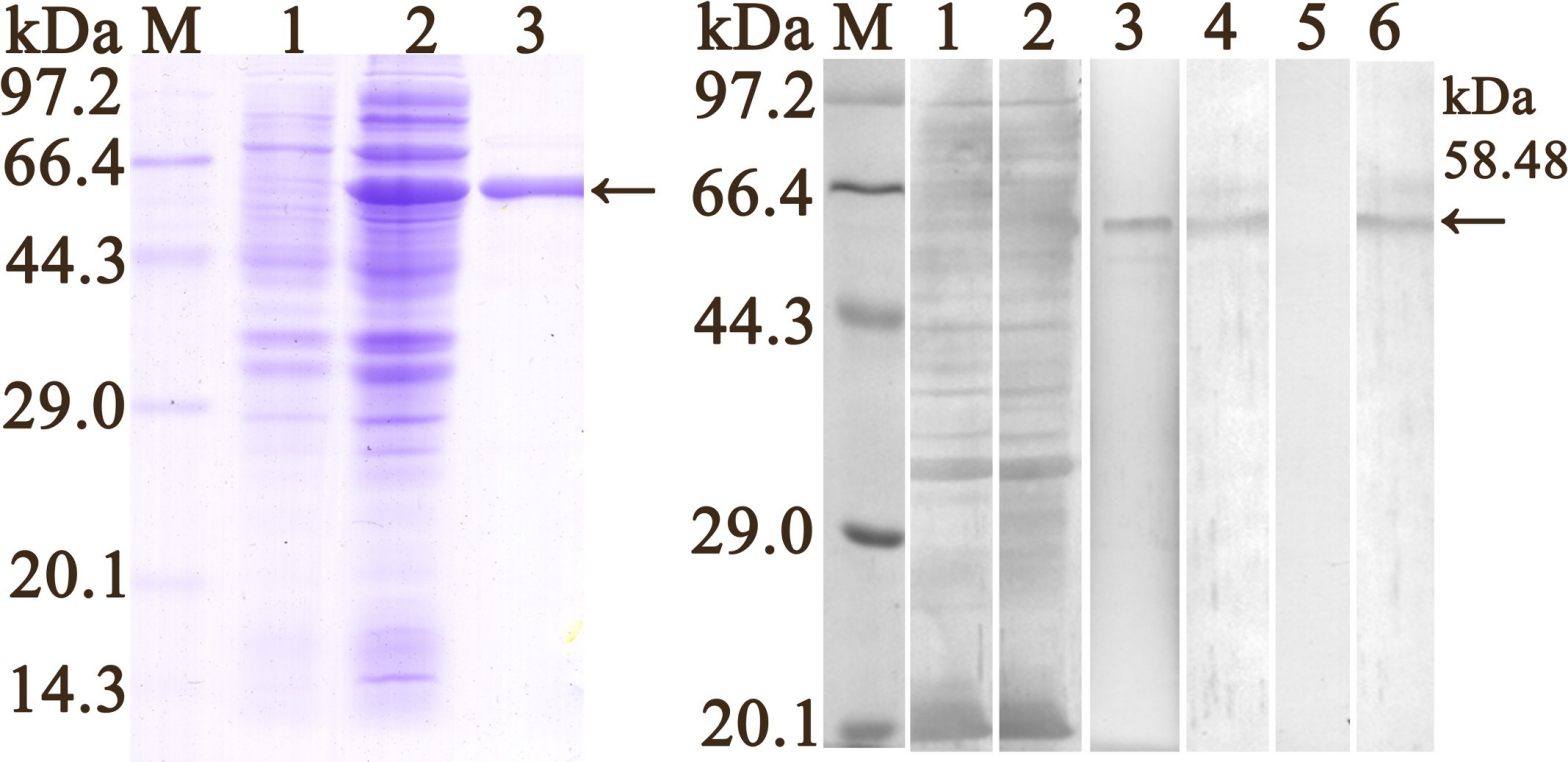
Expression and identification of rTsPKM. **A:** SDS-PAGE analysis of the rTsPKM. Lane M: Protein marker; Lane 1: lysate of recombinant *E*. *coli* incorporating pQE-80L/TsPKM prior to induction; Lane 2: lysate of recombinant *E*. *coli* incorporating pQE-80L/TsPKM after induction; Lane 3: purified rTsPKM. **B.** Western blotting analysis of rTsPKM antigenicity. Lane M: Protein marker; Lane 1: lysates of pQE-80L/TsPKM prior to induction were not recognized by infection serum. Lane 2: lysates of pQE-80L/TsPKM after induction were recognized by infection serum. The purified rTsPKM was recognized by anti-rTsPKM serum (lane 3), infection serum (lane 4) and anti-His tag McAb (lane 6), but not by normal serum (lane 5).

The SDS-PAGE results revealed that the molecular weight (MW) of the fusion protein expressed by the BL21 bacteria carrying pQE-80L/TsPKM was 58.48 kDa, which was consistent with the predicted MW of the TsPKM protein (Fig 3A). In order to evaluate the humoral immune response induced by rTsPKM immunization, the titer of anti-rTsPKM IgG at two weeks after final immunization was detected by ELISA. The results showed that the IgG titer of anti-rTsPKM antibodies reached 1:10^5^ after four immunizations, indicating that rTsPKM had a good antigenicity. Western blotting analysis showed that rTsPKM was recognized by anti-rTsPKM serum, infection serum and anti-his tag monoclonal antibody (McAb) (Fig 3B), but not by pre-immune normal serum.

### Transcription and expression of TsPKM in diverse *T*. *spiralis* stages

**Fig 4:**
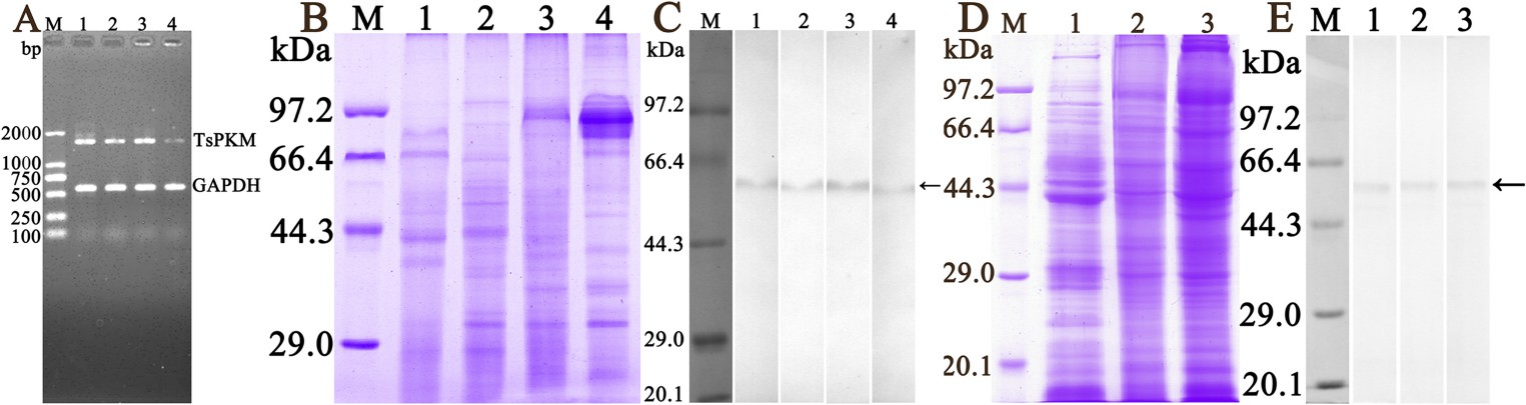
Transcription and expression of TsPKM in different stages of *Trichinella spiralis*. **A:** RT-PCR analysis of TsPKM transcription in diverse stages. Lane M: DNA marker; Lane 1: ML. Lane 2: 6 h IIL. Lane 3: 3 d AW. Lane 4: NBL. **B**: SDS-PAGE analysis of crude proteins of diverse worm stages. Lane M: protein marker. Lane 1: ML soluble protein. Lane 2: IIL soluble protein. Lane 3: 3 d AW soluble protein. Lane 4: NBL soluble protein. **C:** Western blot analysis of crude proteins of diverse worm stages of ML (lane 1), IIL (lane 2), 3 d AW (lane 3) and NBL (lane 4) identified using anti-rTsPKM serum. **D:** SDS-PAGE analysis of ES proteins of ML (lane 1), IIL (lane 2) and 6 d AW (lane 3), Lane M: protein marker. **E:** Western blot analysis of ES proteins of ML (lane 1), IIL (lane 2) and 6 d AW (lane 3) recognized by anti-rTsPKM serum. The recognized native TsPKM with about 58.5 kDa were indicated with arrows.

RT-PCR results revealed that TsPKM gene was transcribed in all various *T*. *spiralis* developmental stage (ML, IIL, 3 d AW and NBL), and the housekeeping gene GAPDH also generated an expected size (570 bp) in all stages of *T*. *spiralis* lifecycle (Fig 4A). On Western blotting analysis, native TsPKM in somatic crude proteins of various worm phases (ML, IIL, 3 d AW and NBL) was identified by anti-rTsPKM serum (Fig 4B and 4C), and the native TsPKM in ES proteins of various phases (ML, IIL and 6 d AW) was also detected by anti-rTsPKM serum (Fig 4D and 4E), indicating that TsPKM was transcribed and expressed at various developmental stages in *T*. *spiralis* lifecycle, and it was a somatic and secretory protein.

### Expression and tissue localization of native TsPKM in various *T*. *spiralis* stages

**Fig 5:**
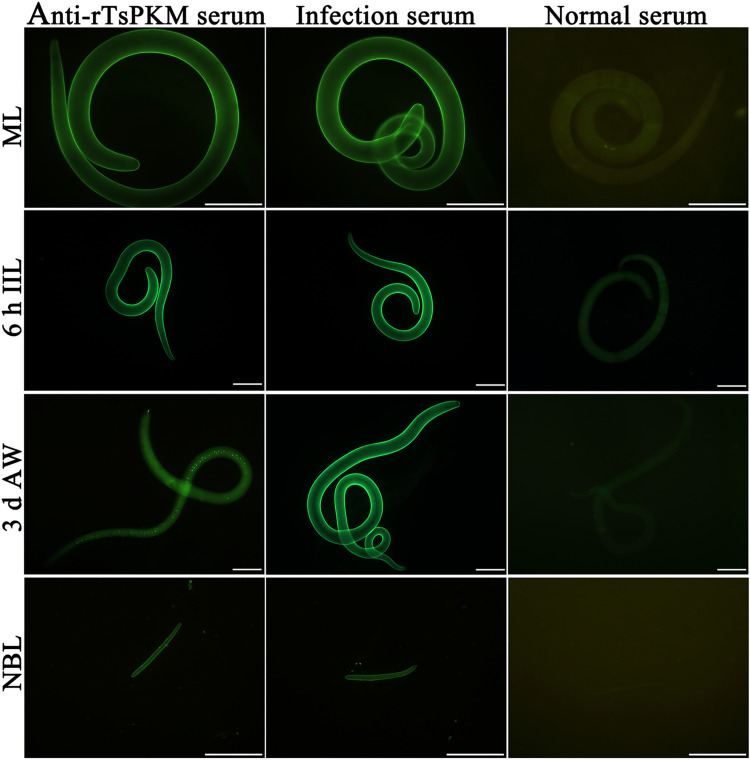
Expression of TsPKM at the cuticle of various *T*. *spiralis* stages by IFT. The whole intact worms were probed by anti-rTsPKM serum, and immune fluorescence staining was observed at the epicuticle of ML, IIL, NBL and the intestine of 3 d AW. But pre-immune normal serum did not recognize any worm components of the nematode. Scale bars: 100 μm.

**Fig 6:**
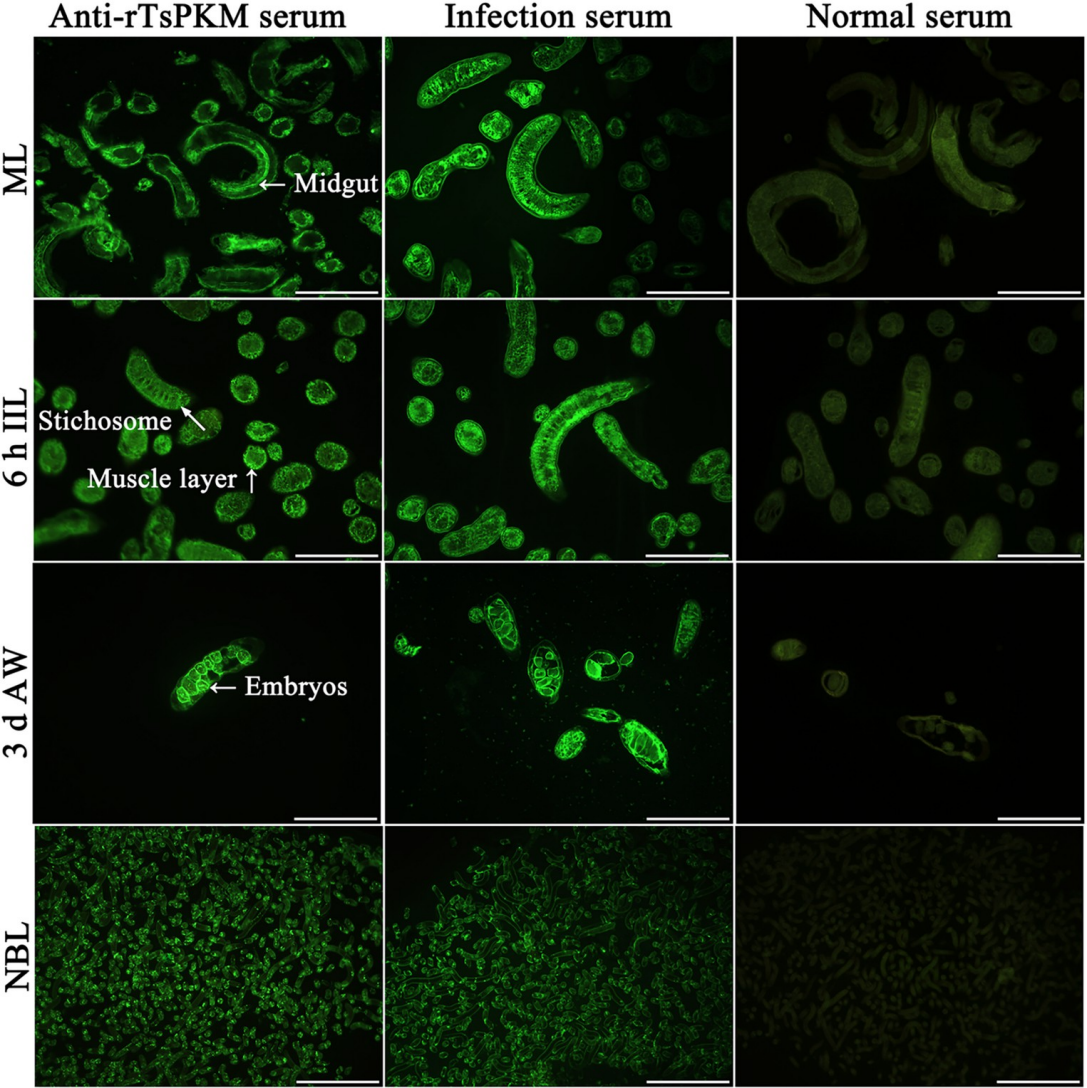
Immunolocalization of TsPKM in worm cross-sections of diverse *T*. *spiralis* stages by IFT with anti-rTsPKM serum. Green fluorescence staining was observed at cuticle, muscle, midgut, stichosome, and female intrauterine embryos. No immunostaining in worm cross-sections was observed by normal serum as a negative control. Scale-bars: 100 μm.

The results of IFT with whole parasites revealed that bright green fluorescence was observed on the out surface of the cuticle of ML, IIL, 3 d AW and NBL using anti-rTsPKM serum and infection serum, but not by normal serum (Fig 5). The results of IFT with worm cross-sections showed that immunostaining was primarily localized in the cuticle, muscle, stichosome, midgut and intrauterine embryos of the female adults (Fig 6).

### Enzymatic activity of rTsPKM

**Fig 7:**
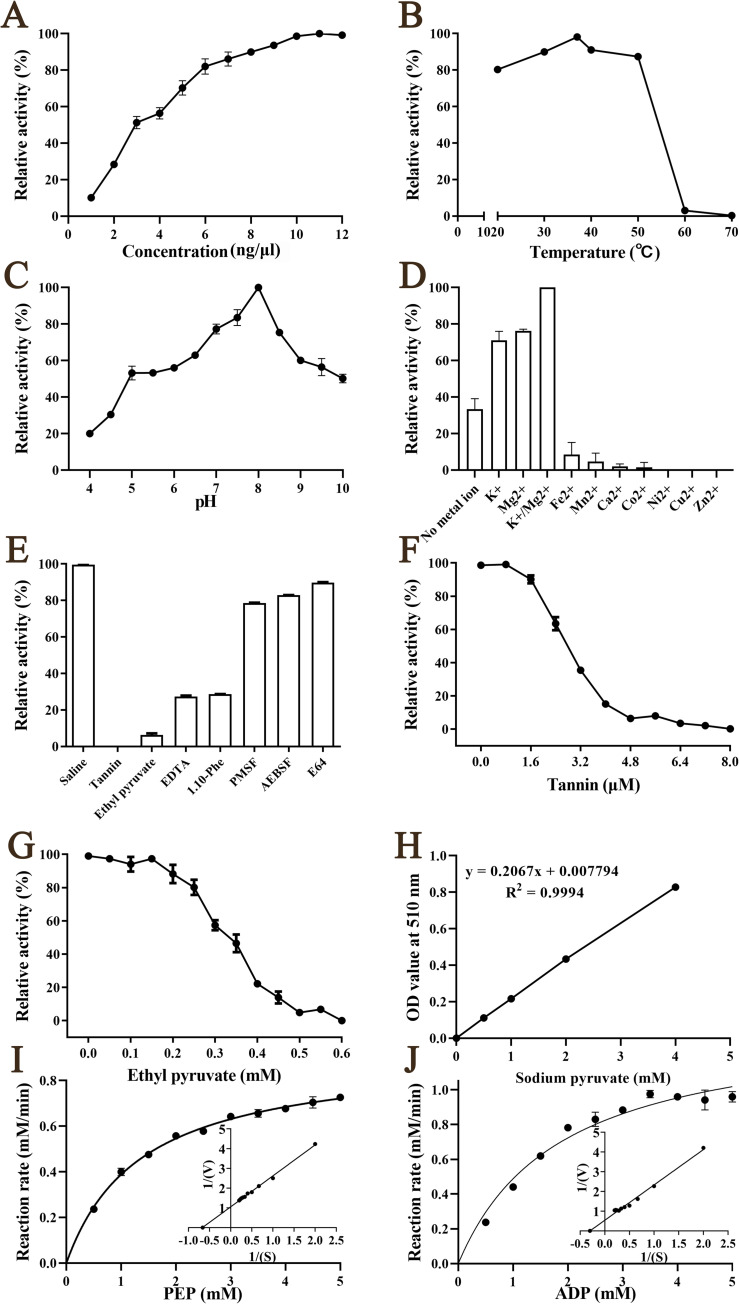
Enzyme activity analysis of rTsPKM. rTsPKM was incubated with 2.5 mm PEP and 1.25mm ADP for 10 min under various conditions. The optimal catalytic conditions of rTsPKM were assessed with various rTsPKM concentrations (1–12 ng/μl), temperatures (20–70°C) and buffer solution with different pH (4–10). **A:** The optimum catalytic concentration of rTsPKM is 10 ng/μl. **B:** The optimum catalytic temperature of rTsPKM is 37°C. **C:** The optimum catalytic pH of rTsPKM is 8.0. **D:** Effects of different metal ions on rTsPKM activity. K^+^ and Mg^2+^ have obvious enhancement role on rTsPKM activity. **E:** Effects of different inhibitors on rTsPKM activity. **F** and **G**: The suppressive role of tannin (**F**) and ethyl pyruvate (**G**) on rTsPKM activity is dose-dependent. **H:** Standard curve of sodium pyruvate. **I:** Michaelis–Menten curve and Lineweaver–Burk of PEP at pH 8.0 and 37°C. **J:** Michaelis–Menten curve and Lineweaver–Burk of ADP at pH 8.0 and 37°C.

The enzymatic activity of rTsPKM gradually increased with elevating rTsPKM concentration, and stabilized at a concentration of 10 ng/μl (Fig 7A). The optimum temperature of rTsPKM for catalyzing the substrate reaction is 37°C, and the optimum buffer pH is 8.0 (Fig 7B and 7C). Metal ions K^+^ and Mg^2+^ enhanced the enzyme activity of rTsPKM whereas Ni^2+^, Fe^2+^, Co^2+^, Ca^2+^, Mn^2+^, Zn^2+^ and Cu^2+^ inhibited the enzyme activity of rTsPKM (Fig 7D). Tannin, ethyl pyruvate, EDTA and 1.10-Phe have obvious inhibitory effects on rTsPKM activity and tannin has the strongest inhibitory effect on rTsPKM activity (Fig 7E). The inhibitory effect of EDTA and 1.10-Phe is because the pyruvate kinase enzyme activity is metal dependent. The suppressive role of tannin (*r* = 0.933, *P* < 0.0001) and ethyl pyruvate (*r* = 0.989, *P* < 0.0001) on rTsPKM activity is dose-dependent (Fig 7F and 7G). The reaction conforms to the simple Michaelise-Menten kinetics (Fig 7H). The kinetic parameter Vmax of PEP is 0.9469 mM/min and the Km value is 1.51 mM (Fig 7I). The kinetic parameter Vmax of ADP is 1.84 mM/min and the Km value is 3.33 mM (Fig 7J).

### Suppression of inhibitors on native TsPKM activity in worm somatic proteins

**Fig 8:**
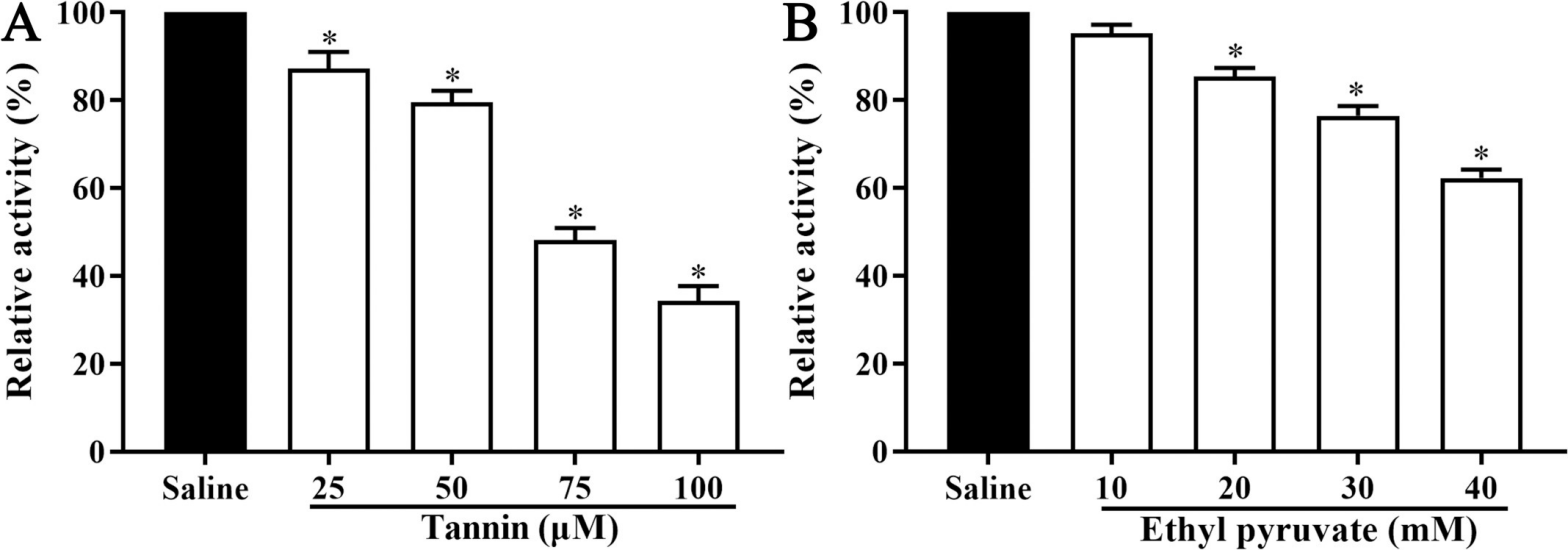
**Suppression of tannin (A) and ethyl pyruvate (B) on native TsPKM enzymatic activity in *T*. *spiralis* muscle larval somatic proteins.** *P < 0.0001 relative to the saline control group.

After the ML were treated with various doses of tannin (25, 50, 75 and 100 μM), enzymatic activity of native TsPKM in ML proteins was reduced by 12.82, 20.45, 51.77 and 65.65% respectively, compared to the saline group without incubation using inhibitors (*F* = 279.731, *P* < 0.0001) (Fig 8A). When ethyl pyruvate (10, 20, 30 and 40 mM) was used, native TsPKM enzymatic activity in ML proteins was reduced by 4.76, 14.64, 23.52 and 37.68%, respectively (*F* = 205.586, *P* < 0.0001) (Fig 8B). The native TsPKM activity in ML protein was notable negative correlation with the doses of tannin (*r* = -0.978, *P* < 0.01) and ethyl pyruvate (*r* = -0.986, *P* < 0.01). When the low doses of inhibitors were used, tannin had better inhibitory effect on native TsPKM activity than ethyl pyruvate. Therefore, tannin was used in the subsequent experiments.

### Reduction of TsPKM expression and activity after silencing TsPKM gene

**Fig 9:**
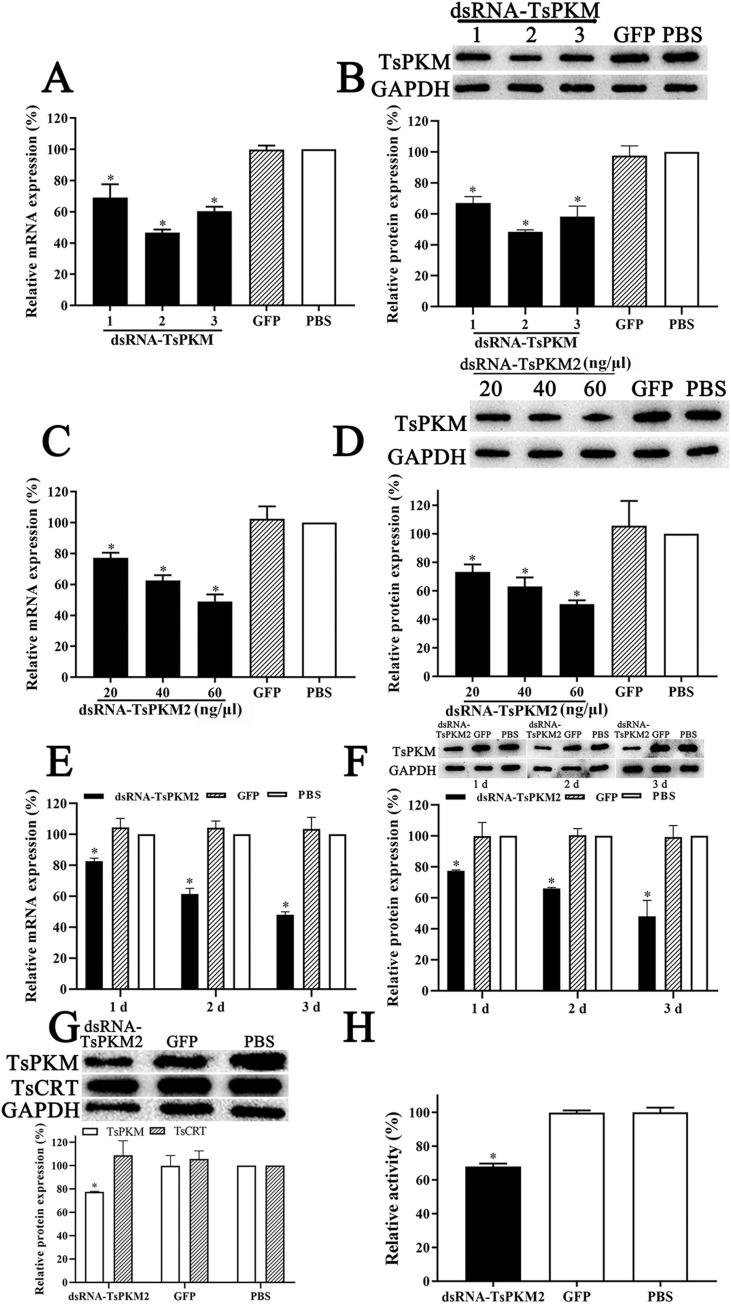
Silencing TsPKM gene suppressed TsPKM expression and enzymatic activity. **A:** TsPKM transcription levels in ML transfected with different dsRNA-TsPKM. **B:** TsPKM expression levels in ML transfected with different dsRNA-TsPKM. **C:** TsPKM transcription levels in ML transfected with various doses of dsRNA-TsPKM2. **D:** TsPKM expression levels in ML transfected with various doses of dsRNA-TsPKM2. **E:** TsPKM transcription levels in ML at 1–3 days after transfection with 60 ng/μl dsRNA-TsPKM2. **F:** TsPKM expression levels in ML at 1–3 days after transfection with 60 ng/μl dsRNA-TsPKM2. **G:** Expression levels of TsPKM and *Trichinella spiralis* calreticulin (TsCRT) in ML treated using dsRNA-TsPKM2. **H:** TsPKM enzyme activity in dsRNA-TsPKM2 treated in ML. *P < 0.05 relative to the PBS group.

After being transfected with 60 ng/μl three dsRNA-TsPKM1, 2, 3 and cultured for 3 days, larval survival of dsRNA-TsPKM1, 2, 3, dsRNA-GFP and PBS group were 93.44, 91.19, 92.63, 92.31 and 93.40%, respectively (χ^2^ = 0.969, *P* > 0.05), demonstrating that electroporation had no obvious effect on larval survival. Moreover, dsRNA-TsPKM2 had the strongest suppressive effect on TsPKM transcription and expression among the three kinds of dsRNA (*F* = 12.190, *P* < 0.01) (Fig 9A and 9B). Therefore, dsRNA-TsPKM2 was used in the following experiment. When the ML were transfected with 20, 40 and 60 ng/μl dsRNA-TsPKM2 and cultured for 3 d, TsPKM mRNA level was reduced by 22.80, 37.30 and 50.91% compared with the PBS group, respectively (*P* < 0.05) (Fig 9C). TsPKM protein expression level was suppressed by 26.81, 36.91 and 49.26% respectively, compared to the PBS group (*P* < 0.05) (Fig 9D). When the ML were transfected with 60 ng/μl dsRNA-TsPKM2 and cultured for 1, 2, 3 d, TsPKM mRNA level was reduced by 17.21, 38.54 and 52.08% compared with the PBS group, respectively (*P* < 0.05) (Fig 9E). TsPKM protein expression level was suppressed by 22.49, 33.85 and 52.03% respectively, compared to the PBS group (*P* < 0.05) (Fig 9F). When the ML were transfected with 60 ng/μl dsRNA-TsPKM2 and cultured for 1 d, *Trichinella spiralis* calreticulin (TsCRT, GenBank: KRY34215.1) protein expression level was not suppressed in ML treated with dsRNA-TsPKM2 (Fig 9G), suggesting that the dsRNA-TsPKM2 is TsPKM-specific. The results of the enzymatic activity assay showed that natural TsPKM enzyme activity in soluble proteins of the ML treated with dsRNA-TsPKM2 was decreased by 26.06% compared to the PBS group (*P* < 0.05) (Fig 9H). Hence, the ML were transfected with 60 ng/μl dsRNA-TsPKM2 and cultured for 3 d in the following experiment.

### Suppression of dsRNA-TsPKM on larval glycometabolism

**Fig 10:**
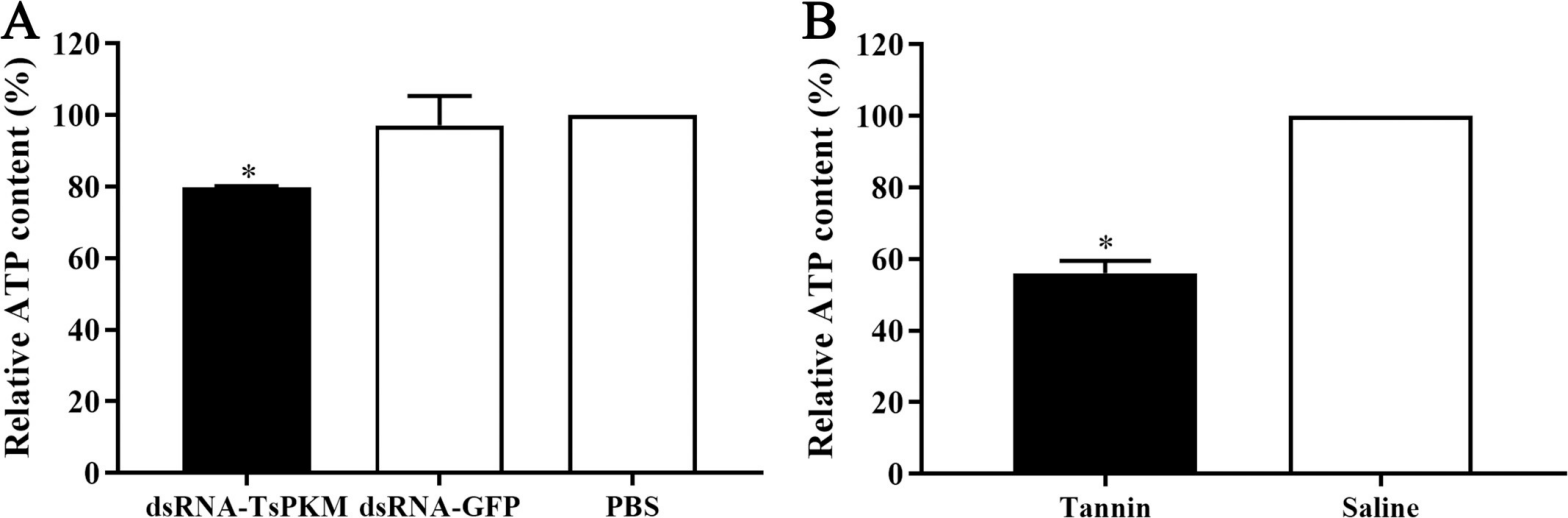
Suppression of dsRNA-TsPKM (A) and tannin (B) on larval ATP content. *P < 0.05 relative to the PBS or saline control group.

After RNAi treatment, the ATP content of the ML from dsRNA-TsPKM, dsRNA-GFP and PBS groups were 4.1416 × 10^−2^, 5.0452 × 10^−2^ and 5.1958 × 10^−2^ μM, respectively. Compared to the GFP and PBS group, ATP content in dsRNA-TsPKM treated ML was decreased by 20.29% (*F* = 10.916, *P* < 0.05) (Fig 10A). After tannin treatment, the ATP content was decreased by 43.99% relative to the saline control group (*t* = 17.427, *P* < 0.05) (Fig 10B).

**Fig 11:**
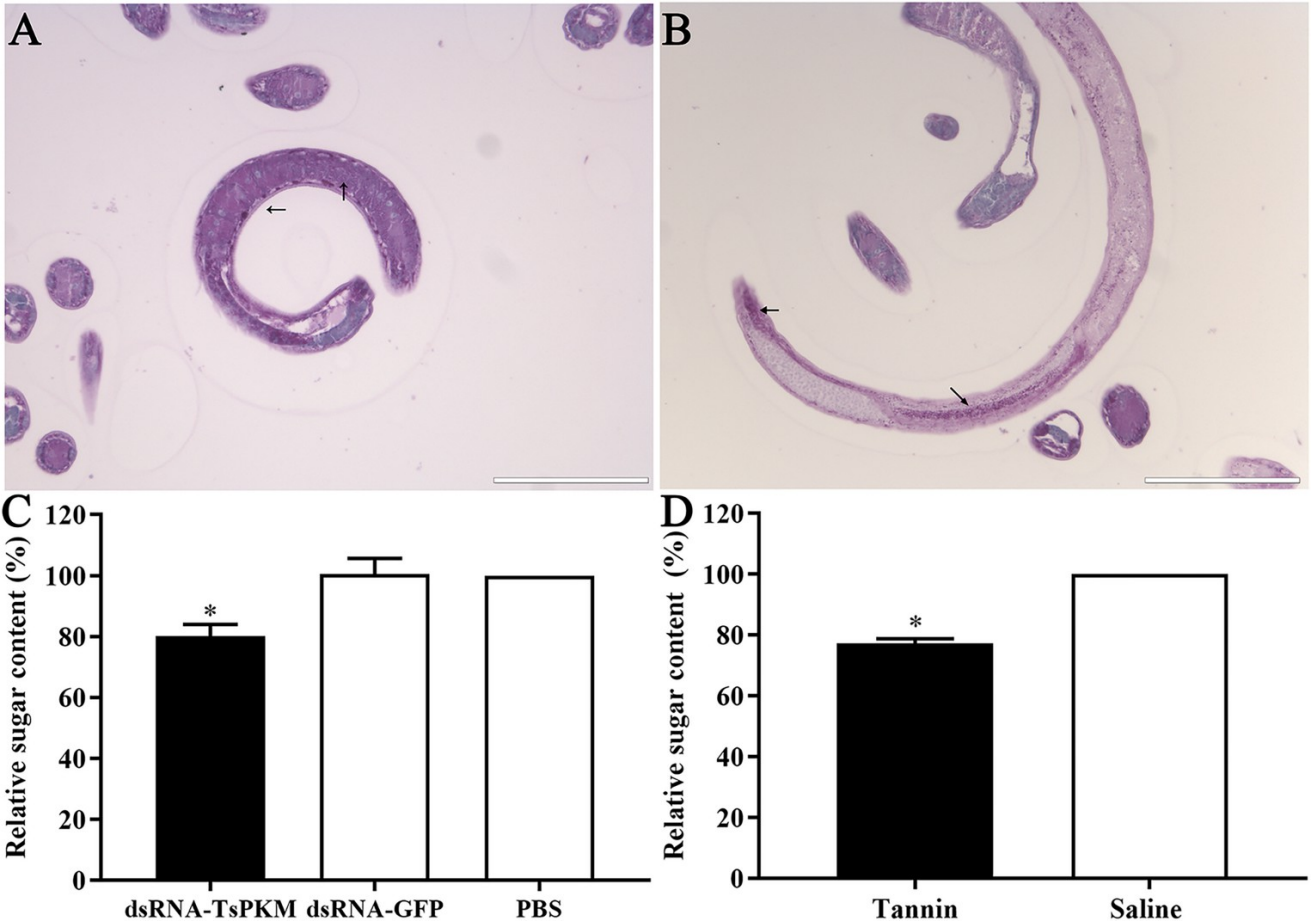
Suppression of dsRNA-TsPKM on *T*. *spiralis* larval glycometabolism. Glycogen is mainly distributed in the muscle larval stichosome (**A**) and around the intestine (**B**). **C:** dsRNA-TsPKM reduced larval total sugar content. **D:** Tannin reduced larval total sugar content. The arrows indicate glycogen. *P < 0.0001 relative to the PBS or saline group. Scale bars: 100 μm.

The results of PAS staining showed that glycogen was mainly distributed around the stichosome and intestine of normal ML (Fig 11A and 11B). The total sugar content in ML of dsRNA-PKM, dsRNA-GFP and PBS groups were 741.8667, 944.5333 and 940.5333 μg respectively. Compared to the GFP and PBS group, total sugar content in ML of dsRNA-PKM group was decreased by 19.86% (*F* = 38.611, *P* < 0.0001). After tannin treatment, the total sugar content in ML was decreased by 22.71% (*t* = 29.302, *P* < 0.0001) (Fig 11C and 11D). The results indicated that TsPKM-specific dsRNA and native inhibitor tannin of pyruvate kinase obviously reduced the sugar content of *T*. *spiralis* worms, suggesting that pyruvate kinase participates in the glycometabolism of the nematode.

**Fig 12:**
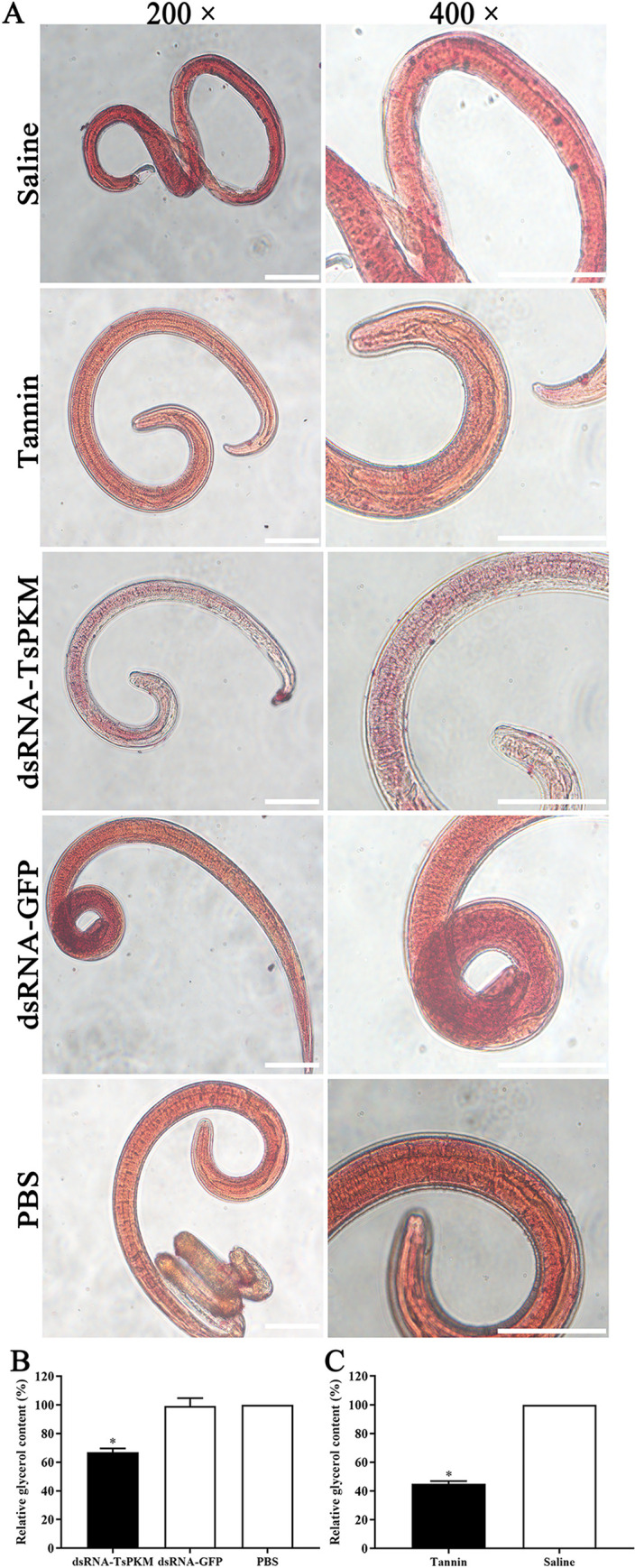
Suppression of dsRNA-TsPKM and tannin on *T*. ***spiralis* larval lipid metabolism A:** Distribution of lipid droplets in different groups of *T*. *spiralis* muscle larvae. Small lipid droplets were distributed all over the muscle larvae, but large lipid droplets were principally localized in the larval intestine and tail. After treatment with dsRNA-TsPKM and tannin, the larval red color became lighter, indicating that larval lipid content of treated larvae with dsRNA-TsPKM and tannin was obviously lower than the control groups. **B:** dsRNA-TsPKM decreased larval lipid content, **C:** tannin decreased larval lipid content. *P < 0.0001 relative to the PBS or saline group. Scale bars: 100 μm.

The result of oil red O staining showed that lipid component in intact muscle larvae was dyed red by oil red O, small lipid droplets were thoroughly distributed in the muscle larvae, but there were large lipid droplets in the intestine and tail of the ML (Fig 12A). The darker the red color, the more the lipid content. After treatment with dsRNA and tannin, the larval red color became lighter, suggesting that larval lipid content of treated larvae with dsRNA and tannin was obviously lower than the control groups. The larval lipid content of dsRNA-TsPKM, dsRNA-GFP and PBS groups were 99.825, 150.7 and 152.075 μg respectively. After silencing of larval TsPKM gene by specific dsRNA, larval lipid content in dsRNA-PKM group was decreased by 33.02%, compared to the PBS group (*F* = 117.450, *P* < 0.0001). After tannin treatment, the larval lipid content was decreased by 54.88% (*t* = 61.662, *P* < 0.0001) (Fig 12B and 12C), suggesting that after larval glycometabolism was inhibited by TsPKM-dsRNA, larval lipid metabolism was also impeded.

### Inhibition of dsRNA TsPKM on the *in vitro* larval molting

**Fig 13:**
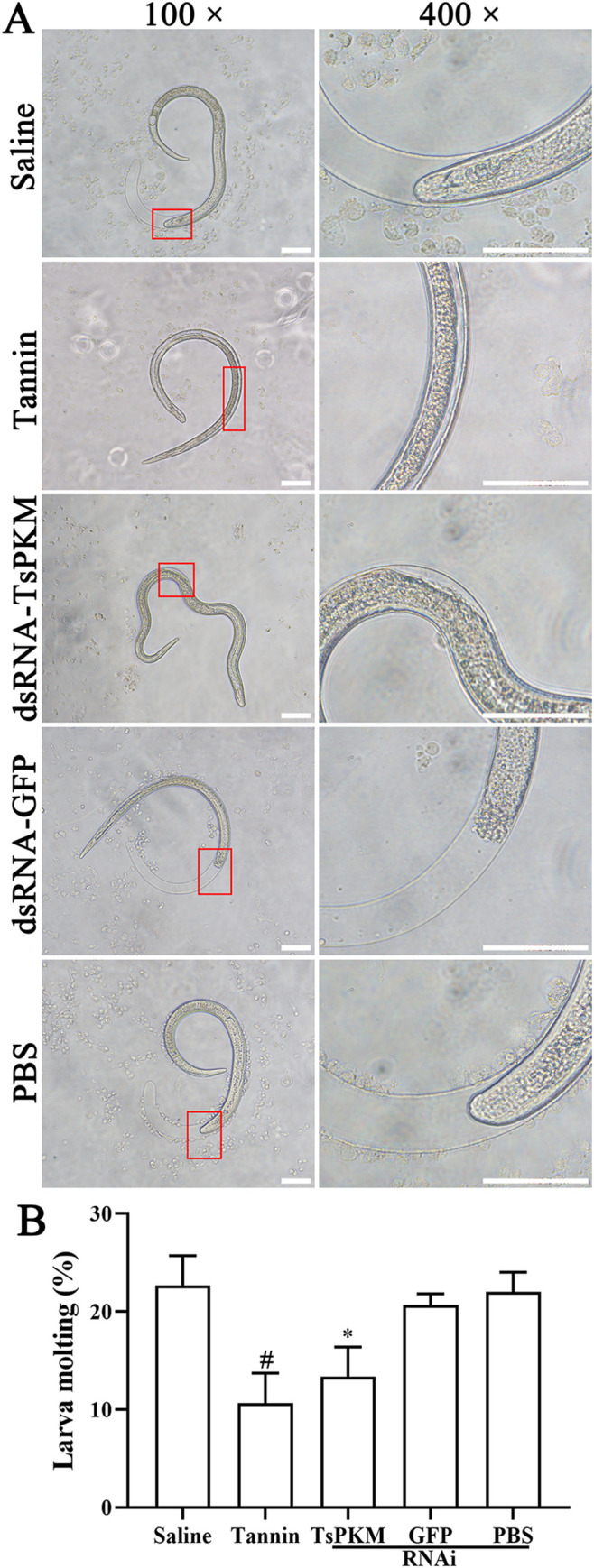
*Trichinella spiralis* larval molting was suppressed by dsRNA-TsPKM in the IIL stage. **A:** RNAi and tannin significantly inhibited the larval molting, and there was no transparent sheath on both the anterior and tail ends. Especially in the tannin group, there was no obvious separation between old and new cuticles. The area in the red box was enlarged for observation. **B:** Molting of *T*. *spiralis* larvae at 3 days after RNAi and tannin treatment. *P < 0.05 relative to the PBS group. ^*#*^*P* < 0.01 relative to the saline group. Scale bars: 200 μm.

The results of the *in vitro* larval molting assay showed that dsRNA-TsPKM and tannin clearly impeded the larval molting and development of *T*. *spiralis* (Fig 13A). Larval molting rate of dsRNA-TsPKM, dsRNA-GFP, PBS, tannin and saline groups was 13.33, 20.67, 22.00, 10.67 and 22.67% respectively. Compared with the PBS group, larval molting in the dsRNA-TsPKM group was decreased by 8.67% (χ^2^ = 3.873, *P* < 0.05). Compared to the saline group, larval molting of the tannin group was decreased by 12% (χ^2^ = 7.776, *P* < 0.01) (Fig 13B). The results suggested that TsPKM also takes part in larval molting and development of the parasite.

### Inhibition of RNAi on the *in vivo* larval development and native TsPKM activity

**Fig 14:**
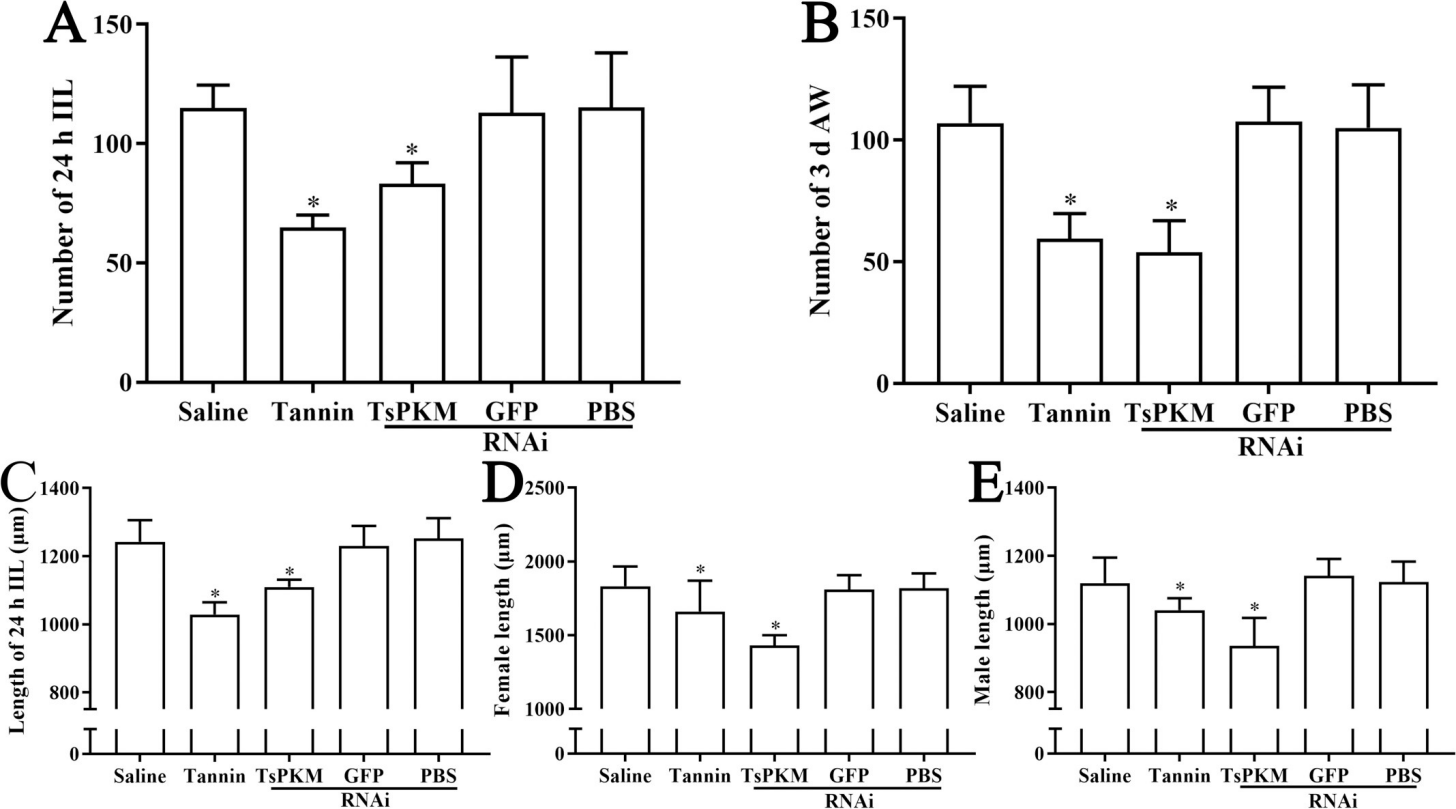
RNAi suppressed the *in vivo* larval development. The numbers of 24 h IIL (**A**) and 3 d AW (**B**) collected from intestine of mice infected with the dsRNA-TsPKM and tannin treated ML. The length of 24 h IIL (**C**) and 3 d female (**D**) and male adult worms (**E**) collected from various groups of infected mice. *P < 0.001 relative to the PBS or saline control group.

TsPKM-specific dsRNA and tannin obviously restrained the larval growth and development in intestine of infected mice, as demonstrated by shorter and smaller worms recovered from the dsRNA-TsPKM and tannin groups (S1 Fig). Compared to the PBS group, the number of recovered IIL and AW of dsRNA-TsPKM treated group was decreased by 27.69 and 48.57%, respectively (*P* < 0.0001). The number of recovered IIL and AW of tannin-treated group evidently declined too, it was reduced by 43.63 and 44.51%, respectively (*P* < 0.0001) (Fig 14A and 14B). After the ML were treated with RNAi and tannin, the length of the IIL and female and male adults from infected mice was also obviously reduced (*F*_IIL_ = 75.806, *F*_female_ = 34.329, *F*_male_ = 37.422, *P* < 0.001). Compared to the PBS group, the length of IIL, female and male adults of the dsRNA group was decreased by 11.37, 21.40 and 16.75%, respectively (*P* < 0.0001). The length of IIL, female and male adults of the tannin group was reduced by 17.14, 9.30 and 7.09% (*P* < 0.0001) (Fig 14C–14E).

**Fig 15:**
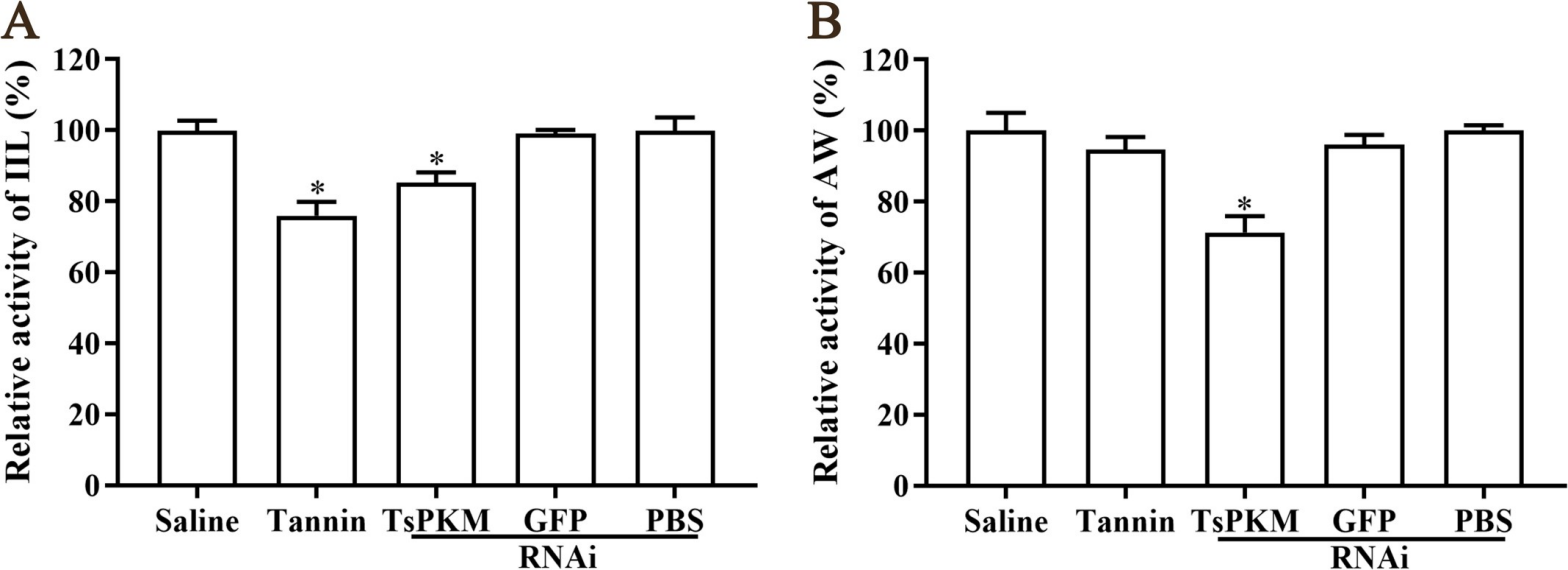
**Inhibition of specific dsRNA-TsPKM on native TsPKM enzymatic activity of 24 h IIL (A) and 3 d AW (B) collected from mice challenged with *T*. *spiralis* ML treated by dsRNA-TsPKM and tannin.** *P < 0.0001 relative to the PBS or saline control group.

Furthermore, RNAi also remarkably suppressed the enzyme activity of native TsPKM of the IIL and AW recovered from infected mice. Compared to the PBS group, native TsPKM enzyme activity of IIL and AW was decreased by 14.76 and 24.10%, respectively (*P* < 0.0001) (Fig 15). The results suggested that specific silencing TsPKM gene evidently impeded the larval invasion, growth and development, also reduced the native TsPKM activity of the parasite in host’s gut.

### Suppression of RNAi on the *in vivo* glycometabolism of adult worms

**Fig 16:**
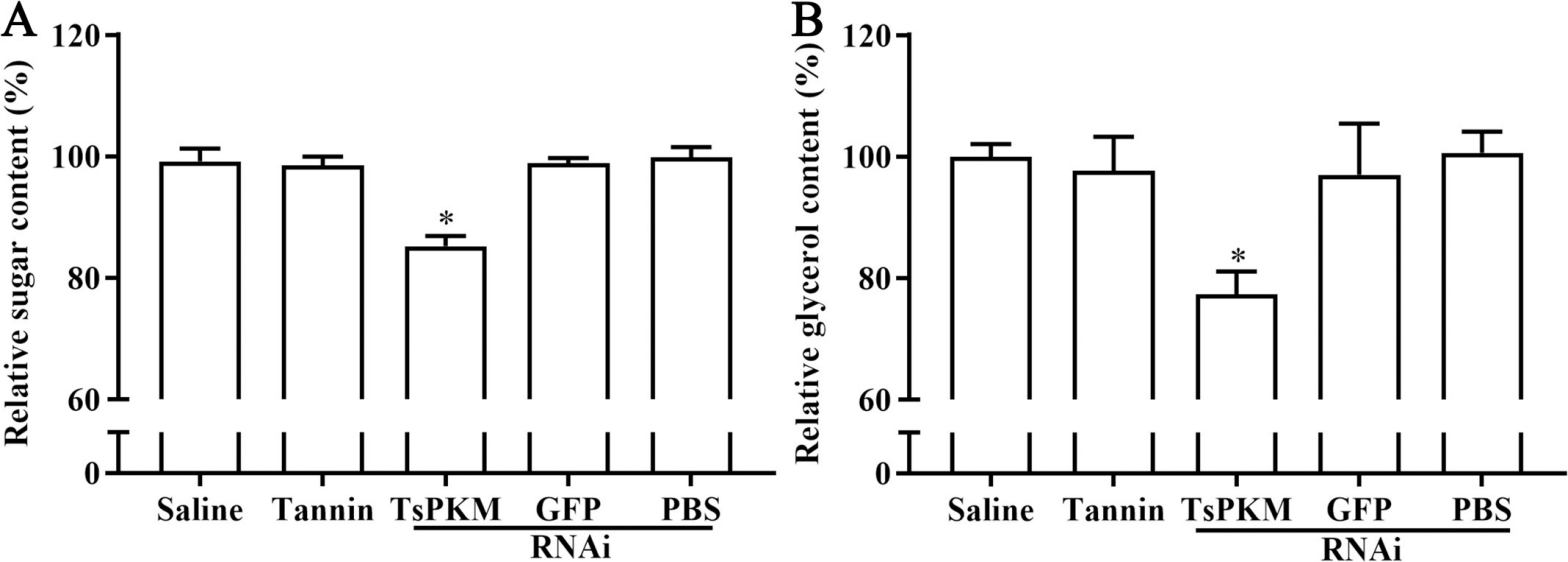
Suppression of dsRNA-TsPKM on sugar and lipid metabolism of *T*. *spiralis* adult worm from infected mice. dsRNA-TsPKM evidently reduced the content of sugar (**A**) and lipid (**B**) in 3 d adult worms. *P < 0.0001 relative to the PBS or saline group.

The result of PAS staining showed that glycogen in 3 d AW was mainly distributed in the muscles, stichosome and around intrauterine embryos (S2 Fig). The results of oil red O staining showed that lipid droplets in adult worms were mainly distributed in intrauterine embryos and around intestine (S3 Fig). The sugar content in 3 d AW of dsRNA-TsPKM, dsRNA-GFP, PBS, tannin and saline groups were 624.7111, 620.2667, 533.1556, 622.9333 and 629.1556 μg respectively (*F* = 46.371, *P* < 0.0001). Compared to the PBS group, the sugar content in 3 d AW of dsRNA-TsPKM group was decreased by 14.59% (*P* < 0.0001). But, the sugar content in 3 d AW of the tannin group was basically unchanged compared to the saline group (*P* > 0.05) (Fig 16A). The lipid contents in 3 d AW of dsRNA-TsPKM, dsRNA-GFP, PBS, saline and tannin groups were 553.1167, 540.2833, 426.6167, 536.6167 and 556.7833 μg respectively (*F* = 10.802, *P* < 0.01). The lipid content of dsRNA-TsPKM treated group was decreased by 23.28% relative to the PBS group (*P* < 0.0001), but the lipid content of tannin treated group had no statistical difference compared to the saline control group (*P* > 0.05) (Fig 16B). The results suggested that PKM-specific dsRNA suppressed the glycometabolism in both larval and adult stages.

## Discussion

At present, albendazole is the first choice for treatment of trichinellosis [73]. Previous studies showed that albendazole has a good killing effect on the intestinal stage of *T*. *spiralis*, but it has a poor killing effect on NBL and ML stages [74]. Additionally, albendazole has a low bioavailability after oral administration with the recorded values being as low as 5%, and such low bioavailability reduced the chance for successful killing of the migrating and encapsulated larvae [75]. Therefore, it is needed to develop new anti-*Trichinella* drugs. Glycolysis is found to be a good therapeutic target for some parasitic infection, such as *Schistosoma japonicum* [12], *Plasmodium* [15], and so on. Pyruvate kinase as drug target for different parasitic diseases could be verified by gene knockout, RNAi and inhibitors.

In the current study, the complete sequence of TsPKM gene was cloned and expressed in prokaryotic expression system. Sequence analysis revealed that TsPKM had an identity of 98.71, 98.71, 98.53, 98.52, 98.34, 98.34 and 97.79% with pyruvate kinase of the 7 encapsulated *Trichinella* species/genotypes (*T*. *nativa*, *T*. *patagoniensis*, *T*. *murrelli*, *Trichinella* T8, *T*. *nelsoni*, *T*. *britovi* and *Trichinella* T6), and it had an identity of 88.89, 88.21 and 88.21% with pyruvate kinase from 3 non-encapsulated *Trichinella* species (*T*. *pseudospiralis*, *T*. *papuae* and *T*. *zimbabwensis*). Phylogenetic tree revealed that a monophyletic group of the genus *Trichinella* was well supported. Bioinformatics analysis revealed that the TsPKM belonged to transferases. It had two functional domains of pyruvate kinase, and the enzyme active site. After expression, solubility analysis showed that rTsPKM existed in both supernatant and inclusion body, and the expression level in supernatant was higher. rTsPKM in supernatant was easier to be folded and aggregated correctly, and its protein structure was closer to natural TsPKM, which was more likely to have the catalytic activity function of natural TsPKM [76]. Immunization of mice with rTsPKM induced a specific anti-rTsPKM antibody response, the serum titer of specific anti-rTsPKM IgG reached 1: 10^5^, demonstrating that rTsPKM had a good immunogenicity.

The results of RT-PCR and Western blot showed that TsPKM was transcribed and expressed in various *T*. *spiralis* stages. The IFT results showed that TsPKM was mainly distributed in the epidermis, muscle cells, stichosome, and intestine and around the embryos. Western blotting results also indicated that rTsPKM was recognized by anti-rTsPKM serum and infected serum, but not by normal mouse serum; natural TsPKM was found in ES proteins of different *T*. *spiralis* stages. Previous study showed that enzymes of glucose metabolism pathways such as pyruvate kinase exist in ML, 3 d AW and NBL, but the expression level was obviously different in various worm stages, which was related to the different parasitic positions of *T*. *spiralis* at different stages and the changes of aerobic metabolism and anaerobic metabolism [77]. The pyruvate kinase activity of the ML from freshly killed mice was evidently increased after 60 min-digest and saline incubation for 40 min, suggesting that the activation processes of *T*. *spiralis* infective ML larvae are stimulated upon liberation of the larvae from the nurse cell inside the host stomach, and the metabolic switch from anaerobic metabolism of infective ML stage to aerobic metabolism was found in the enteral stages [78]. The result of the current study was consistent with the expression of PKM in other parasites, which was mainly distributed in tissues with high energy metabolism, such as muscle and rapid embryo proliferation [13,79]. Our results indicated that TsPKM was expressed in various *T*. *spiralis* stages and it was a secretory protein, demonstrated that TsPKM is a necessary protein in *T*. *spiralis* lifecycle and might participate in larval molting, sugar metabolism, development and reproduction of this parasite [72]. The results suggested that secretory TsPKM could be exposed directly to host’s immune system and trigger the production of anti-TsPKM antibodies during *T*. *spiralis* infection [80,81].

The enzyme activity of rTsPKM was detected by 2,4-dinitrophenylhydrazine chromogenic method, the results showed that rTsPKM expressed in this study had the catalytic activity of natural pyruvate kinase. In the buffer system, metal ions K^+^ and Mg^2+^ significantly enhanced the enzyme activity of rTsPKM, suggesting that K^+^ and Mg^2+^ played an indispensable role in the activity of pyruvate kinase. K^+^ and Mg^2+^ hardly affect the structure of pyruvate kinase, however the domain can be reversed under the action of K^+^ and Mg^2+^, which made the active site of pyruvate kinase more exposed [82]. For different concentrations of ethyl pyruvate and tannin, the inhibition roles were increased with the increase of inhibitor concentration. rTsPKM enzyme activity was completely inhibited by 0.60 mM ethyl pyruvate and 8 μM tannin. In tumor tissues, the proliferation of tumor cells was through K433 site of PKM2, and tannin selectively inhibits the pyruvate kinase activity of PKM2, rather than protein kinase activity and PKM2 expression, to suppress colorectal cancer cell proliferation, tannin is a promising PKM2 inhibitor for prevention of colorectal cancer [83] Ethyl pyruvate also has a good inhibitory effect on PKM, it is safe for red blood cells and easy to penetrate the blood-brain barrier [56]. In *Trypanosoma brucei*, 5 mM ethyl pyruvate incubated with trypanosomes for 3 h can completely inhibit the proliferation of trypanosomes and kill trypanosomes [56]. Tannin and ethyl pyruvate inhibited the enzymatic activity of pyruvate kinase in *Trichinella spiralis* and other parasites, but the difference of effective inhibitory concentrations may be related with the size and parasitic site of the parasites.

RNAi has been widely used for study on the gene function of various parasites [60]. In order to further verify the function of TsPKM in the sugar metabolism, growth and development of this nematode, RNAi and tannin were used in this study. After treatment with60 ng/μl of dsRNA-PKM2 for 3 days and 100 μM tannin for 2 hours, the catalytic activity of TsPKM, ATP content, total sugar content and lipid content in ML distinctly decreased, suggesting that RNAi and tannin suppressed larval sugar metabolism, reduced ATP production. Molting is the most significant feature of intestinal larval development in *T*. *spiralis* lifecycle [84]. In the present study, the *in vitro* IIL molting was evidently inhibited by RNAi and tannin treatment. The results of animal challenge experiments showed that the number and length of 24 h IIL and 3 d AW and the content of total sugar and lipid in 3 d AW in dsRNA TsPKM group were significantly reduced and inhibited. Furthermore, RNAi also remarkably suppressed the enzyme activity of native TsPKM of the IIL and AW recovered from infected mice. The results further confirmed that TsPKM played an important role in the larval molting, metabolism, growth and development of this parasite. In previous studies on other parasites, inhibition of pyruvate kinase led to the decline of ATP level, metabolic disorder and obvious inhibition of larval growth and development of larvae. The inhibition of pyruvate kinase expression in *Giardia canis* using specific hammerhead ribozyme decreased pyruvate kinase enzyme activity and impaired growth and development of *G*. *canis* trophozoites [85]. Knockout of PYK1 and PYK2 genes by CRISPR/Cas9 technology completely hindered the growth and caused the death of *Toxoplasma gondii* [16]. The results suggested that pyruvate kinase might be a potential candidate drug target against *Trichinella* infection.

In conclusion, a novel TsPKM was cloned and expressed, its biological characteristics and roles in sugar metabolism, larval molting and development of *T*. *spiralis* were investigated in this study. The results showed that TsPKM was transcribed and expressed at various *T*. *spiralis* developmental stages, mainly localized at cuticle, muscle layer, stichosome, intestine and around the embryos. The rTsPKM had the native enzymatic activity of pyruvate kinase to catalyze the reaction of PEP and ADP. The silencing of TsPKM gene by TsPKM-specific dsRNA significantly reduced the expression levels and enzyme activity of native TsPKM in the larvae, and RNAi also suppressed larval molting, sugar metabolism, growth and development of this parasite. The results indicated that TsPKM is an obligatory enzyme in *T*. *spiralis* lifecycle; it was involved in larval molting, sugar metabolism and development, and may be regarded as a potential candidate drug target against *T*. *spiralis* infection.

## Supporting information

S1 Fig Morphology of the IIL and adult worms recovered from mice challenged with *T*. ***spiralis* ML treated with dsRNA-TsPKM and tannin.** Scale bars: 200 μm.
(JPG)

S2 Fig Distribution of glycogen in cross-sections of 3 d adult worms from different groups of infected mice. **A:** Saline group. **B:** Tannin group. **C and D**: dsRNA-TsPKM group. **E:** dsRNA-GFP group. **F:** PBS group. Glycogen of 3 d AW is mainly distributed in muscles, stichosome and around intrauterine embryos. Black arrows indicate the glycogen. Scale bars: 100 μm.
(JPG)

S3 Fig Distribution of lipid droplets in whole adult worms of different groups of infected mice at 3 days post infection. Lipid droplets were mainly distributed in intrauterine embryos and around intestine of adult worms. Scale bars: 200 μm.
(JPG)
