# Supplementary figures and images for: Characterization of a novel pyruvate kinase from Trichinella spiralis and its participation in sugar metabolism, larval molting and development
Source: PLoS Negl Trop Dis. 2022 Oct 31;16(10):e0010881. doi: 10.1371/journal.pntd.0010881 (PMC9621426; doi:10.1371/journal.pntd.0010881)

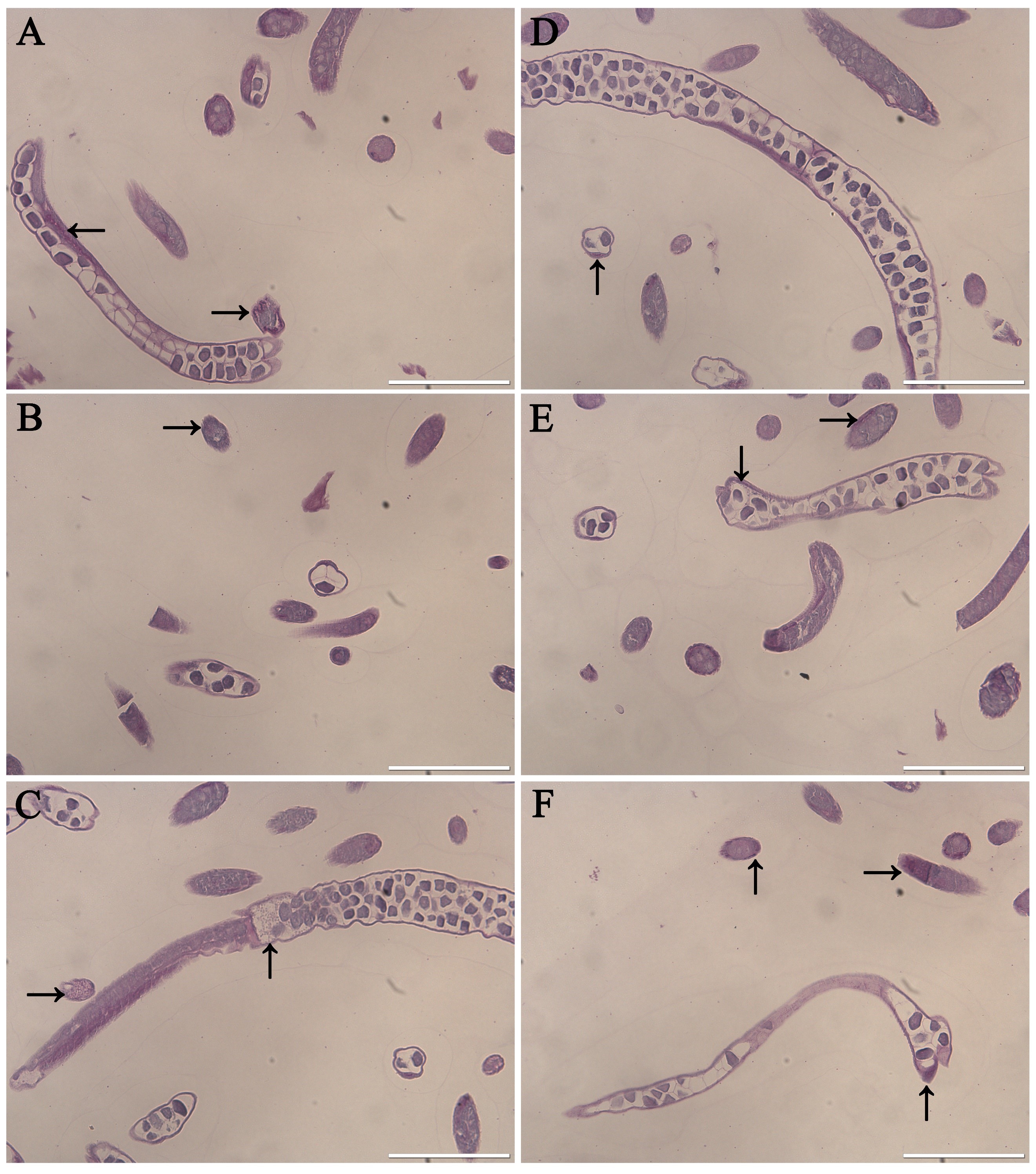

Supplement: S2 Fig — A: Saline group. B: Tannin group. C and D: dsRNA-TsPKM group. E: dsRNA-GFP group. F: PBS group. Glycogen of 3 d AW is mainly distributed in muscles, stichosome and around intrauterine embryos. Black arrows indicate the glycogen. Scale bars: 100 μm. (JPG) [file pntd.0010881.s002.jpg]
